# Magnetic resonance imaging characteristics of brain metastases in small cell lung cancer

**DOI:** 10.1002/cam4.6206

**Published:** 2023-06-08

**Authors:** Dan Zhu, Yongjia Shao, Zhangwei Yang, Ailan Cheng, Qian Xi, Xiaohua Liang, Shuguang Chu

**Affiliations:** ^1^ Department of Radiology, Shanghai East Hospital Tongji University School of Medicine Shanghai China; ^2^ Department of Oncology, Shanghai Huashan Hospital Fudan University School of Medicine Shanghai China

**Keywords:** brain metastases, diffusion‐weighted imaging, magnetic resonance imaging, small cell lung cancer, T1‐weighted contrast enhancement

## Abstract

**Background:**

Lung is the most common primary site of brain metastases (BMs). For different pathological types of BMs have some similar characteristics, it is still a challenge to confirm the origin based on their characteristics directly. BMs of small cell lung cancer (SCLC) have favorable therapeutic expectations due to their high sensitivity to radiotherapy. This study sought to identify unique characteristics of BMs in SCLC, aiming to assist in clinical decision‐making.

**Methods:**

Patients diagnosed with BMs of lung cancer who received radiotherapy from January 2017 to January 2022 were reviewed (*N* = 284). Definitive diagnosis of BMs of SCLC was reached for 36 patients. All patients underwent head examination using magnetic resonance imaging. The number, size, location, and signal characteristics of lesions were analyzed.

**Results:**

There were 7 and 29 patients with single focus and non‐single focus, respectively. Ten patients had diffuse lesions, and the remaining 26 patients had a total of 90 lesions. These lesions were divided into three groups according to size: <1, 1–3, and >3 cm (43.33%, 53.34%, and 3.33%, respectively). Sixty‐six lesions were located in the supratentorial area, primarily including cortical and subcortical lesions (55.56%) and deep brain lesions (20%). Moreover, 22 lesions were located in the infratentorial area. According to diffusion‐weighted imaging and T1‐weighted contrast enhancement, the imaging characteristics were classified into six patterns. Hyperintensity in diffusion‐weighted imaging and homogeneous enhancement was the most common pattern of BMs in SCLC (46.67%), while partial lesions showed hyperintensity in diffusion‐weighted imaging without enhancement (7.78%).

**Conclusions:**

The manifestations of BMs in SCLC were multiple lesions (diameter: 1–3 cm), hyperintensity in diffusion‐weighted imaging, and homogeneous enhancement. Interestingly, hyperintensity in diffusion‐weighted imaging without enhancement was also one of the characteristics.

## 1 INTRODUCTION

Lung cancer is one of the most malignant types of tumors, and associated with high morbidity and mortality; it is mainly divided into two subtypes, namely small cell lung cancer (SCLC) and non‐small cell lung cancer (NSCLC).^1^ SCLC accounts for 14% of all lung cancers cases, and 10% of patients initially diagnosed with SCLC develop brain metastases (BMs).^2^, ^3^ Distinguishment of the different subtypes of BMs is necessary for clinical decision‐making. Owing to their high sensitivity to radiotherapy, BMs of SCLC are associated with a favorable prognosis. Therefore, prompt identification can lead to early intervention, which improves the quality of life of patients.^4^, ^5^

Magnetic resonance imaging (MRI) offers multi‐directional imaging and high resolution of soft tissue; hence, it is a useful tool in the identification of BMs.^6^ It can be used for disease diagnosis, evaluation of treatment response, monitoring for tumor recurrence, and the identification of new metastases. Current guidelines recommend using head MRI for the detection of BMs in patients with advanced lung cancer.^7^ Therefore, it is crucial to evaluate the morphology and characteristics of BMs through MRI. In addition, analysis of the number, size, location, and signal characteristics of BMs may provide new directions for qualitative analysis. Diffusion‐weighted imaging (DWI) is a functional imaging method used to characterize the diffusion status of water molecules.^8^ Studies have shown that DWI is valuable in the diagnosis of brain tumors.^9^, ^10^, ^11^ T1‐weighted (T1WI) contrast enhancement is an indicator of blood supply to tumors, and tumor enhancement represents destruction of the blood–brain barrier. The combination of these two techniques may provide more information for the identification of metastatic tumors.

Multiple lesions with peritumoral edema are recognized as the characteristics of BMs, but it is not sufficient to identify the source of the primary tumor. Meanwhile, nodules with edema are also characteristic of some infectious lesions, such as brain abscesses. Therefore, other sequences (such as DWI and T1WI contrast enhancement) are necessary for routine auxiliary diagnosis. Furthermore, different types of BMs require different treatment modalities; thus, the distinction of these types is essential. BMs of SCLC are typically treated with chemotherapy or radiotherapy. In NSCLC, early‐stage BMs can be treated with surgery; chemotherapy is the best option for patients in whom the opportunity for surgery is missed.^12^, ^13^ Moreover, the distinction of BMs can assist physicians regarding the treatment of patients presenting with neurological symptoms at their first admission to hospital. Despite their high sensitivity to radiation therapy, BMs in SCLC are linked to a poor prognosis. Hence, prompt identification of their features is helpful for early diagnosis and the administration of treatment. Therefore, identification of the characteristics of BMs in SCLC is important.

In this study, we evaluated the imaging characteristics of 36 patients with BMs of SCLC. The number, size, location, and MRI signal characteristics of lesions were analyzed. The purpose of this study was to improve the diagnostic sensitivity for BMs of SCLC and accurately identify patients with BMs of SCLC who present with neurological symptoms at their initial admission to hospital.

## 2 MATERIALS AND METHODS

### 2.1 Patients

We reviewed 284 patients diagnosed with BMs who were treated at the radiation department of Shanghai East Hospital (Shanghai, China) from January 2017 to January 2022. Among them, 36 patients were diagnosed with BMs of SCLC. The diagnosis of lung cancer was confirmed pathologically by bronchoscopy or surgery; cerebral metastases were detected based on biopsy or cerebrospinal fluid examination. We collected the clinical characteristics of all patients, including age, sex, smoking status, etc. The study was approved by the ethics committee of Shanghai East Hospital and conformed to the Declaration of Helsinki. Due to the retrospective nature of this study, the requirement for informed consent was waived.

### 2.2 MRI acquisitions

All patients underwent head MRI examinations in the radiology department. MRI images were captured with a 3.0‐T MR device (M750w; GE Healthcare) and a 1.5‐T MR device (PHILIPS Achieva). All images included the following sequences: axial T1WI images; T2WI; T2 flair images, followed by tri‐planar T1W FSE sequences after intravenous administration of a single 0.1 mg/kg bolus dose of contrast agent (gadopentetate dimeglumine; Magnevist; Bayer Schering Pharma). DWI was acquired by a single‐shot spin echo planar imaging sequence. The specific parameters were as follows: T1WI: repetition time (TR): 4000 ms; echo time (TE): 112 ms; field of view (FOV): 230 × 230 mm; T2WI: TR: 4000 ms; TE: 112 ms; FOV: 230 × 230 mm; DWI: TR: 13,700 ms; TE: 85 ms; FOV: 224 × 224 mm; and thickness/slice gap: 2/0.6.

## 3 RESULTS

We analyzed the imaging and clinical characteristics of 36 patients with BMs of SCLC. The male‐to‐female ratio was 7:2, and the mean age of patients was 67 years (range: 47–78 years). Among them, there were 17 and 19 smoking and non‐smoking patients, respectively.

According to the number of lesions, we divided patients into single focus and non‐single focus groups. Patients in the non‐single focus group were further divided according to the number of lesions (i.e., 1, 2–3, 4–5, 6–10, >10 lesions). These subgroups included 7, 13, 5, 4, and 7 patients, respectively, accounting for 19.45%, 36.11%, 13.89%, 11.11%, and 27.78% of cases, respectively (Table 1). Of these 36 patients, 10 patients had diffuse lesions. The remaining 26 patients had a total of 90 lesions; the size, location and peritumoral edema of these lesions were analyzed (Table 2). In terms of size, lesions with diameter <1 and 1–3 cm accounted for 43.33% and 53.34% of cases, respectively. On the contrary, lesions with diameter >3 cm only accounted for 3.33% of cases.

**TABLE 1:** Number of brain metastases in SCLC.

Number of lesions	Number of patients	Proportion (%)
1	7	19.45
2–3	13	36.11
4–5	5	13.89
6–10	4	11.11
>10	10	27.78
Total	36	100

**TABLE 2:** MRI characteristics of brain metastases in SCLC.

Characteristics	Number	Proportion (%)
Size	90	100
<1 cm	39	43.33
1–3 cm	48	53.33
>3 cm	3	3.33
Deep brain	18	20
Centrum semiovale	4	4.44
Thalamus	3	3.33
Periventricular	8	8.89
Brainstem	2	2.22
Callosum	1	1.11
Cortical or subcortical	50	55.56
Frontal lobe	23	25.56
Parietal lobe	17	28.33
Temporal lobe	7	11.67
Occipital lobe	3	3.33
Cerebellum	22	24.44
Peritumoral edema	40	44.44

Abbreviations: MRI, magnetic resonance imaging; SCLC, small cell lung cancer.

Concerning the location of the lesions, shallow position was defined as the cortical or subcortical areas, while deep brain referred to the areas of the periventricular, centrum semiovale, callosum, brainstem, and other deeper areas in the brain. A total of 66 lesions were located in the supratentorial area; of those, cortical/subcortical and deep brain lesions accounted for 55.56% and 20% of cases, respectively. The most common sites of cortical and subcortical lesions were the frontal and parietal lobes, accounting for 25.56% and 28.33% of lesions respectively; the periventricular area was the most common location of lesions in the deep brain. Other rare positions included the temporal lobe, occipital lobe, centrum semiovale, thalamus, brainstem, and callosum. In addition, 24.45% of lesions were identified in the cerebellum (located in the infratentorial area). For peritumoral edema, this characteristic was found in 44.44% of total lesions.

**FIGURE 1:**
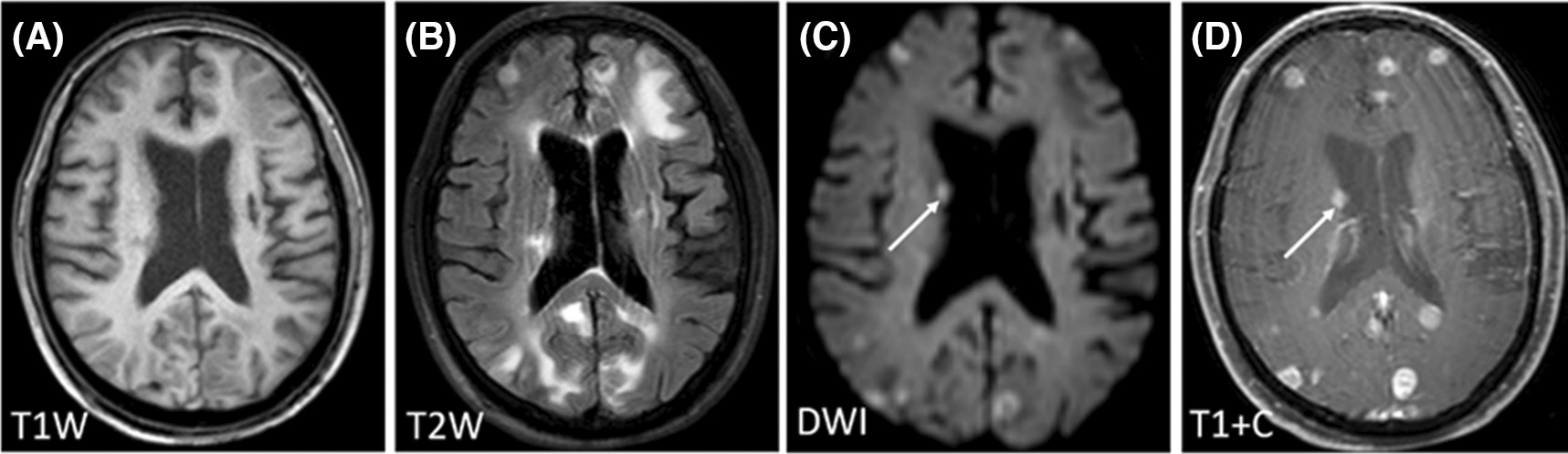
Pattern I of brain metastases in SCLC. Pattern I: A 77‐year‐old woman without history of smoking was diagnosed with BMs 1 year after diagnosis of SCLC. The lesions were numerous and located in supratentorial and infratentorial areas. The lesion (white arrow) was located in the right periventricular, with hypointensity in T1WI (A), hyperintensity in T2WI and DWI (B, C), and homogeneous enhancement (D). BMs, brain metastases; DWI, diffusion‐weighted imaging; SCLC, small cell lung cancer.

**FIGURE 2:**
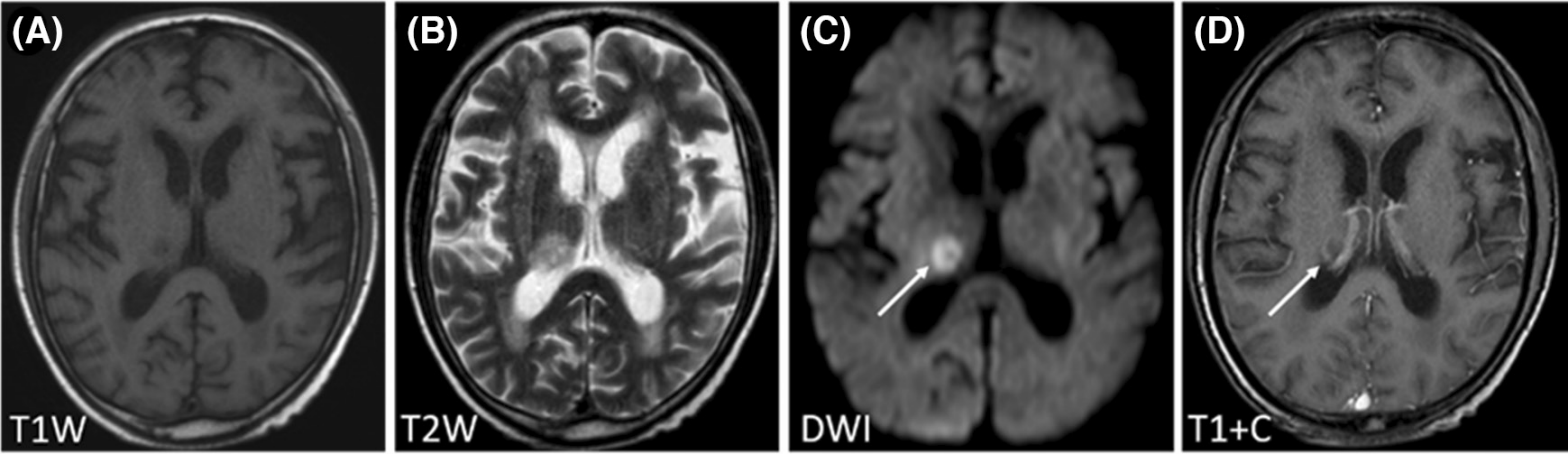
Pattern II of brain metastases in SCLC. Pattern II: A 67‐year‐old man was diagnosed with BMs of SCLC for the first time. The patient had four lesions. One lesion (white arrow) was located in the right thalamus, with hypointensity in T1WI (A), hyperintensity in T2WI and DWI (B, C), and ring enhancement (D). BMs, brain metastases; DWI, diffusion‐weighted imaging; SCLC, small cell lung cancer.

**FIGURE 3:**
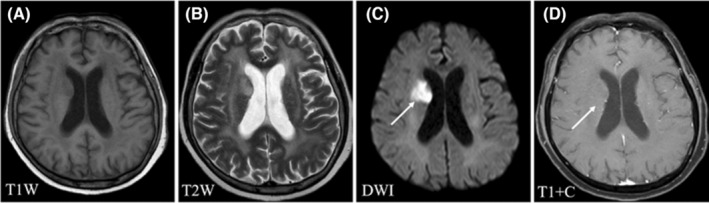
Pattern III of brain metastases in SCLC. Pattern III: A 71‐year‐old man without history of smoking was diagnosed with SCLC. A follow‐up head MRI examination revealed the presence of three intracranial lesions located in the right periventricular, callosum, and left centrum semiovale. One lesion (white arrow) was located in the right periventricular, with hypointensity in T1WI (A), hyperintensity in T2WI and DWI (B, C), and no enhancement (D). Cerebral infarction was initially diagnosed. Finally, BMs of SCLC were confirmed by cerebrospinal fluid examination and imaging findings during follow‐up. BMs, brain metastases; DWI, diffusion‐weighted imaging; MRI, magnetic resonance imaging; SCLC, small cell lung cancer.

**FIGURE 4:**
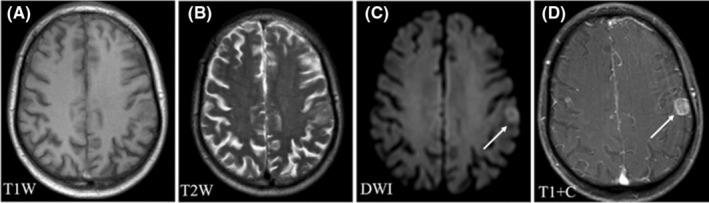
Pattern IV of brain metastases in SCLC. Pattern IV: A 78‐year‐old man was diagnosed with BMs of SCLC. The patient had numerous lesions located in the supratentorial and infratentorial areas. One lesion (white arrow) was located in the frontal lobe, with hypointensity in T1WI (A), hyperintensity in T2WI and DWI (B, C), and homogeneous enhancement (D). BMs, brain metastases; DWI, diffusion‐weighted imaging; SCLC, small cell lung cancer.

**FIGURE 5:**
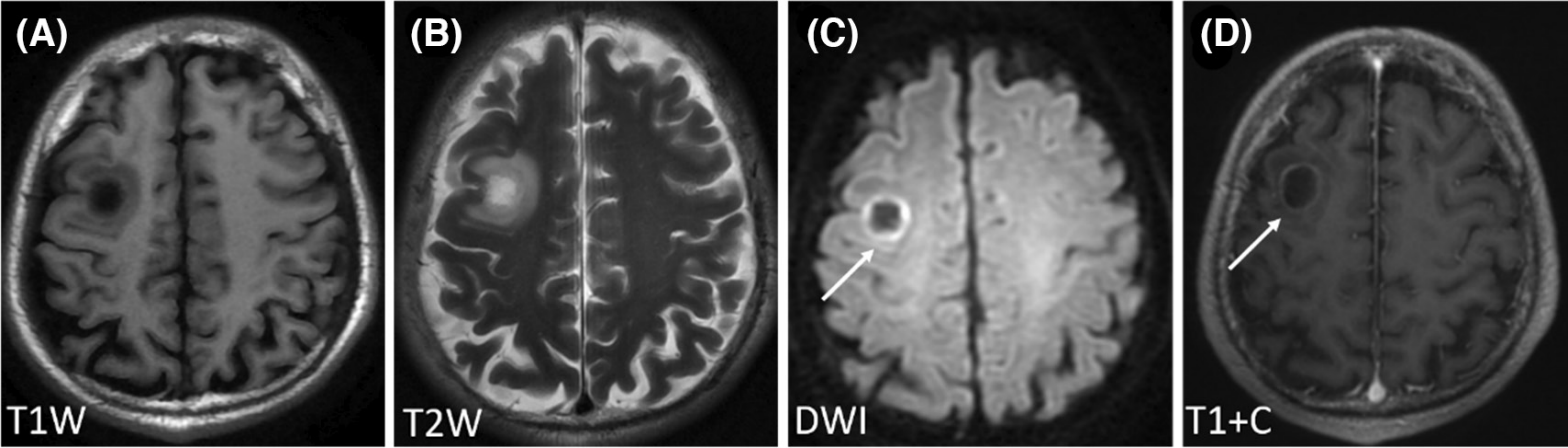
Pattern V of brain metastases in SCLC. Pattern V: A 62‐year‐old man with a history of smoking was diagnosed with BMs of SCLC. The patient had nine lesions. One lesion (white arrow) was located in the right frontal lobe, with hypointensity in T1WI (A), hyperintensity in T2WI (B), ring hyperintensity in DWI (C) and ring enhancement (D). BMs, brain metastases; DWI, diffusion‐weighted imaging; SCLC, small cell lung cancer.

**FIGURE 6:**
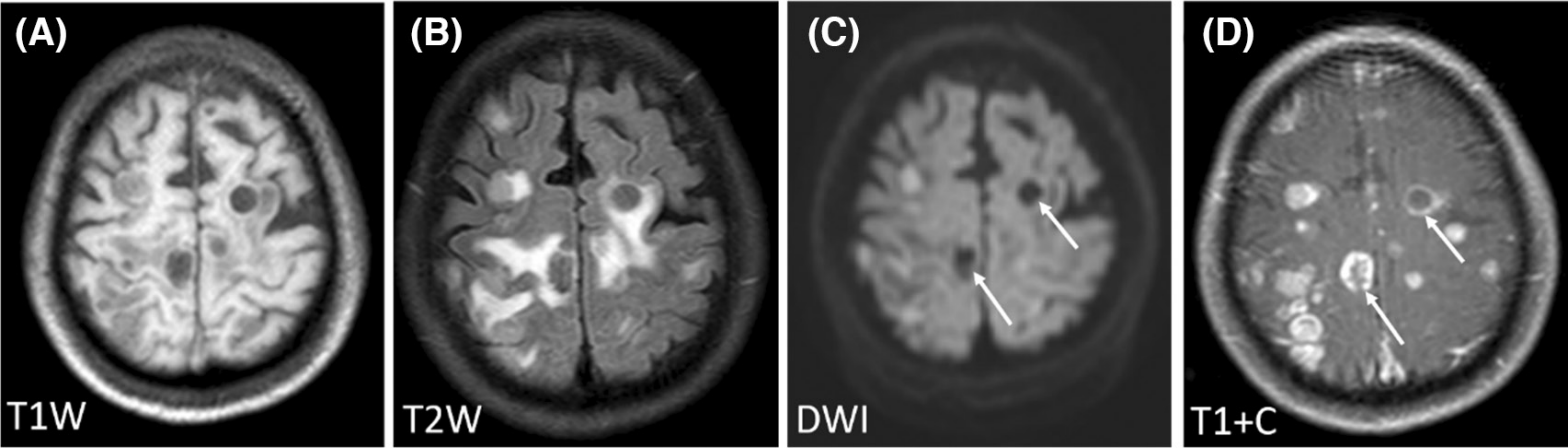
Pattern VI of brain metastases in SCLC. Pattern VI: A 77‐year‐old woman without history of smoking was diagnosed with BMs of SCLC. The patient had numerous lesions located in the supratentorial and infratentorial areas. The lesions (white arrows) were located in the frontal lobe and centrum semiovale, with hypointensity in T1WI (A), hypointensity in T2WI (B), hypointensity in DWI (C) and homogeneous and ring enhancement (D). BMs, brain metastases; DWI, diffusion‐weighted imaging; SCLC, small cell lung cancer.

Regarding the signal characteristics, since most lesions showed plain/negative signal on T1WI and positive signal on T2WI, which had no specificity. Therefore, we did not analyze them in the study. Hyperintensity in DWI was observed for 92.21% of the lesions, and lesions with homogeneous enhancement, ring enhancement, and no enhancement accounted for 62.22%, 30%, and 7.78% of cases, respectively. We further divided these lesions into six patterns according to the signal characteristics of DWI and T1WI contrast enhancement (Table TABLE 3). The first pattern was characterized by hyperintensity in DWI and homogeneous enhancement; this was the most common pattern and accounted for the highest proportion of cases (46.67%) (Figure 1). The second pattern was characterized by hyperintensity in DWI and ring enhancement, accounting for 16.67% of cases (Figure 2). Moreover, 7.78% of the lesions exhibited hyperintensity in DWI but no enhancement (Figure 3). In addition, some lesions showed ring hyperintensity in DWI with homogeneous enhancement or ring hyperintensity with ring enhancement; these patterns accounted for 7.78% and 13.33% of cases, respectively (Figures 4 and 5). The last pattern involved hypointensity in DWI and homogeneous enhancement, accounting for 7.78% of cases (Figure 6).

**TABLE 3:** Six patterns of brain metastases in SCLC.

Pattern	DWI	T1‐weighted contrast enhancement	Number	Proportion (%)
I	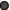	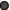	42	46.67
II	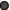	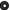	15	16.67
III	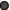	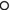	7	7.78
IV	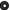	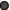	7	7.78
V	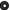	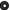	12	13.33
VI	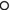	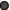	7	7.78

*Note*: 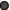: homogeneous hyperintensity; 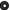: ring hyperintensity; 3.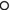: hypointensity.

Abbreviation: DWI, diffusion‐weighted imaging.

## 4 DISCUSSION

In this study, we evaluated the MRI characteristics of BMs in SCLC. We found that multiple lesions, diameter of 1–3 cm, hyperintensity in DWI, and homogeneous enhancement were important characteristics in this setting. The most frequent locations of BMs were the frontal lobe, parietal lobe, and cerebellum, though deep brain areas may also be involved. Interestingly, hyperintensity in DWI without enhancement is also a noteworthy characteristic.

Lung cancer is divided into SCLC and NSCLC; the former type accounts for 14% of all lung cancer cases.^14^ Due to the large difference in the incidence of SCLC and NSCLC, research conducted thus far focused mainly on the latter type. This is also true for research on BM of SCLC and NSCLC. Numerous imaging characteristics of NSCLC have been established based on radionics and machine deep learning.^15^, ^16^ However, due to the low incidence of BMs of SCLC, their features have not been comprehensively evaluated.

A total of 284 patients diagnosed with BMs of lung cancer and treated with radiotherapy were retrospectively investigated. Among those, 36 patients were diagnosed with BMs of SCLC: 32 and four cases were confirmed by lung and brain biopsies, respectively. SCLC is a highly malignant and aggressive type of lung neuroendocrine carcinoma.^14^ Studies have shown that patients with SCLC are at high risk of developing BMs. In addition, with the continuous improvement of treatment measures and extension of patient survival, the rate of BMs of SCLC has been gradually increased.^17^ Therefore, the identification of BM characteristics of SCLC may be helpful for early diagnosis and treatment. Moreover, a clear understanding of the imaging characteristics of BMs in different primary malignancies may be useful in the management of patients with ambiguous or multiple primary tumors.

The present study found that the incidence of BMs in SCLC was higher in males versus females, while the commonly observed age of onset was middle and older age. These findings are consistent with the prevalence trends of lung cancer. Moreover, 80.55% of patients presented with multiple lesions, which is a basic feature of metastasis. This characteristic was noted in BMs of SCLC. Similar to previous studies, the present analysis revealed that BMs of SCLC tended to have a smaller diameter than those of NSCLC.^10^ In the 36 patients examined in this investigation, 96.67% of the lesions had a diameter <3 cm. The average diameter of lesions was 1.2 cm. The reasons responsible for the size of these lesions may be related to the morphology of primary lesions of lung cancer. Lesions measuring >3 cm were characterized by intratumoral bleeding; these lesions showed hyperintensity on T1WI. Therefore, we hypothesized that intratumoral hemorrhage caused the increase in lesion diameter. We concluded that BMs in SCLC tended to be “multiple and smaller in size” compared with those in NSCLC.

According to previous research, brain anatomy has a certain relationship with BM. The cerebellum, gray matter junction area, and watershed regions are predominantly affected.^18^ In addition, the location of BMs was also closely related to that of primary tumors.^19^, ^20^ The analysis revealed that more lesions were located in the cortex and subcortex than in deeper areas of the brain. Moreover, the frontal and parietal lobes were the most common supratentorial locations of BMs. A previous study illustrated that the BMs of SCLC were not randomly distributed, and the cerebellum was the most common position.^21^ Our research yielded consistent findings. The frontal lobe, parietal lobe, and cerebellum were the most common locations of BMs in SCLC, possibly due to their anatomy and ample blood flow. However, the importance of some other uncommon locations (e.g., brainstem and callosum) should not be ignored. Therefore, the diagnosis should be based on clinical symptoms and other examinations. A comprehensive understanding of the characteristics of lesions in different sites may improve the accuracy of diagnosis. Peritumoral edema is often considered a feature of BMs, and in less than half of our cases, peritumoral edema was present, which may be related to the relatively small number of lesions we included. In addition, different manifestations can occur in the same patient and may be related to rapid lesion progression.

Studies showed that DWI was of great value in the evaluation of BMs.^22^ Furthermore, the current guidelines also recommend DWI as an effective tool in the diagnosis of BMs.^6^ Several studies have demonstrated that most BMs of SCLC show hyperintensity on DWI or reduction of the apparent diffusion coefficient (ADC).^9^, ^10^, ^12^, ^23^ In general, lower ADC values have been associated with poor prognosis.^24^, ^25^ T1WI contrast enhancement represents destruction of the blood–brain barrier, and is an important indicator in the diagnosis of BMs.^6^ However, the specific imaging characteristics of BMs in SCLC have not been determined yet. Therefore, we combined DWI with T1WI contrast enhancement to investigate the imaging characteristics of such lesions.

Among the 90 lesions studied, 92.22% showed hyperintensity on DWI. Therefore, DWI appears to be a reliable indicator in the diagnosis of BMs of SCLC. Hyperintensity and ring hyperintensity in DWI and T1WI contrast enhancement were the most common features, which can be manifested in a variety of combinations. The pattern of manifestation may be determined by several factors, such as the pathological and molecular typing of tumors, as well as changes occurring after systemic therapy.^26^ Traditionally, ring enhancement was considered one of the specific features of metastases. However, we found that homogeneous enhancement was the most common feature of BMs in SCLC. Possible reasons for this observation include the small diameter of lesions, and cell membranes had bigger quantities, as well as the lesions were not easy to necrosis.

We should also pay close attention to the atypical pattern which manifests as hyperintensity on DWI without enhancement. It was difficult to distinguish this type from cerebral infarction this distinction may require rich clinical experience. Hence, the accumulation of relevant experience is necessary to avoid misdiagnosis in future clinical practice. Lesions that showed hypointensity in DWI and visible T1WI contrast enhancement were also detected. Although misdiagnosis of this type was less likely, the presence of such lesions highlighted the importance of enhanced MRI for patients with a history of SCLC.

The present study had several limitations. Firstly, the sample size analyzed in our study was small. Secondly, we did not compare the characteristics of BMs in SCLC with those of BMs in NSCLC. In a subsequent study involving more cases, we will conduct a comparative analysis and investigate additional features of BMs of SCLC based on deep learning.

## 5 CONCLUSIONS

In conclusion, we found that the lesions of BMs in SCLC were relatively smaller than those of NSCLC, with an average diameter of 1.2 cm. Moreover, there was a tendency for multiple lesions in BMs, with the frontal lobe, parietal lobe, and cerebellum being the most common locations. Hyperintensity on DWI or reduction of the ADC can be features of a differential diagnosis. In addition, most lesions exhibited homogeneous enhancement. Particularly, hyperintensity on DWI without enhancement was also a feature of BMs in SCLC, which should also be taken into consideration. The present results may improve the diagnostic sensitivity for BMs of SCLC and assist in the accurate identification of such patients who present with neurological symptoms at their initial admission to hospital.

## AUTHOR CONTRIBUTIONS

**Dan Zhu:** Writing – original draft (lead). **Yongjia Shao:** Methodology (lead). **Zhangwei Yang:** Software (supporting). **Ailan Cheng:** Methodology (equal); software (equal). **Qian Xi:** Investigation (lead); visualization (lead); writing – review and editing (equal). **Xiaohua Liang:** Resources (lead); writing – review and editing (equal). **Shuguang Chu:** Funding acquisition (supporting); project administration (lead); supervision (lead).

## FUNDING INFORMATION

This work was supported by the Discipline Construction of Health System in Pudong New Area, Shanghai (PWzbr2022‐14).

## CONFLICT OF INTEREST STATEMENT

The authors have no conflicts of interest.

## ETHICS STATEMENT

The study conformed to the Declaration of Helsinki. The research protocol was approved by the Institutional Reviewer Board: Shanghai East Hospital (Shanghai, China). The study was conducted in accordance with relevant code of ethics and regulations. Due to the retrospective nature of this study, the requirement for informed consent was waived.

## CONSENT FOR PUBLICATION

Not applicable.

## Data Availability

Availability of data and materials The data used to support the findings of this study are available from the corresponding author upon request.

## References

[1] Siegel RL , Miller KD , Fuchs HE , Jemal A . Cancer statistics, 2021. CA Cancer J Clin. 2021;71:7‐33. 33433946 10.3322/caac.21654

[2] Zhu Y , Cui Y , Zheng X , Zhao Y , Sun G . Small‐cell lung cancer brain metastasis: from molecular mechanisms to diagnosis and treatment. Biochim Biophys Acta Mol Basis Dis. 2022;1868:166557. 36162624 10.1016/j.bbadis.2022.166557

[3] Stelzer KJ . Epidemiology and prognosis of brain metastases. Surg Neurol Int. 2013;4:S192‐S202. 23717790 10.4103/2152-7806.111296 PMC3656565

[4] Fox BD , Cheung VJ , Patel AJ , Suki D , Rao G . Epidemiology of metastatic brain tumors. Neurosurg Clin N Am. 2011;22:1‐6. 21109143 10.1016/j.nec.2010.08.007

[5] Seute T , Leffers P , ten Velde GP , Twijnstra A . Detection of brain metastases from small cell lung cancer: consequences of changing imaging techniques (CT versus MRI). Cancer. 2008;112:1827‐1834. 18311784 10.1002/cncr.23361

[6] Ellingson BM , Bendszus M , Boxerman J , et al. Consensus recommendations for a standardized brain tumor imaging protocol in clinical trials. Neuro Oncol. 2015;17:1188‐1198. 26250565 10.1093/neuonc/nov095 PMC4588759

[7] Kaufmann TJ , Smits M , Boxerman J , et al. Consensus recommendations for a standardized brain tumor imaging protocol for clinical trials in brain metastases. Neuro Oncol. 2020;22:757‐772. 32048719 10.1093/neuonc/noaa030 PMC7283031

[8] Le Bihan D . Looking into the functional architecture of the brain with diffusion MRI. Nat Rev Neurosci. 2003;4:469‐480. 12778119 10.1038/nrn1119

[9] Incesu L , Abdullayev S , Ozturk M , Aslan K , Gunbey HP . Role of apparent diffusion coefficient measurement in differentiating histological subtypes of brain metastasis of lung cancer. Rev Assoc Med Bras. 1992;2022(68):1318‐1323. 10.1590/1806-9282.20220630 PMC9575026 36228265

[10] Müller SJ , Khadhraoui E , Neef NE , Riedel CH , Ernst M . Differentiation of brain metastases from small and non‐small lung cancers using apparent diffusion coefficient (ADC) maps. BMC Med Imaging. 2021;21:70. 33858368 10.1186/s12880-021-00602-7 PMC8048287

[11] Jung WS , Park CH , Hong CK , Suh SH , Ahn SJ . Diffusion‐weighted imaging of brain metastasis from lung cancer: correlation of MRI parameters with the histologic type and gene mutation status. AJNR Am J Neuroradiol. 2018;39:273‐279. 29301782 10.3174/ajnr.A5516 PMC7410588

[12] Bozdağ M , Er A , Çinkooğlu A . Histogram analysis of ADC maps for differentiating brain metastases from different histological types of lung cancers. Can Assoc Radiol J. 2021;72:271‐278. 32602365 10.1177/0846537120933837

[13] Pangua C , Rogado J , Serrano‐Montero G , et al. New perspectives in the management of small cell lung cancer. World J Clin Oncol. 2022;13:429‐447. 35949427 10.5306/wjco.v13.i6.429 PMC9244973

[14] Barnholtz‐Sloan JS , Sloan AE , Davis FG , Vigneau FD , Lai P , Sawaya RE . Incidence proportions of brain metastases in patients diagnosed (1973 to 2001) in the metropolitan Detroit cancer surveillance system. J Clin Oncol. 2004;22:2865‐2872. 15254054 10.1200/JCO.2004.12.149

[15] Jünger ST , Hoyer UCI , Schaufler D , et al. Fully automated MR detection and segmentation of brain metastases in non‐small cell lung cancer using deep learning. J Magn Reson Imaging. 2021;54:1608‐1622. 34032344 10.1002/jmri.27741

[16] Wu MY , Zhang EW , Strickland MR , et al. Clinical and imaging features of non‐small cell lung cancer with G12C KRAS mutation. Cancer. 2021;13:3572. 10.3390/cancers13143572 PMC8304953 34298783

[17] Schild SE , Sio TT , Daniels TB , Chun SG , Rades D . Prophylactic cranial irradiation for extensive small‐cell lung cancer. J Oncol Pract. 2017;13:732‐738. 29125923 10.1200/JOP.2017.026765

[18] Takano K , Kinoshita M , Takagaki M , et al. Different spatial distributions of brain metastases from lung cancer by histological subtype and mutation status of epidermal growth factor receptor. Neuro Oncol. 2016;18:716‐724. 26519739 10.1093/neuonc/nov266 PMC4827044

[19] Schroeder T , Bittrich P , Kuhne JF , et al. Mapping distribution of brain metastases: does the primary tumor matter? J Neurooncol. 2020;147:229‐235. 32065345 10.1007/s11060-020-03419-6 PMC7075842

[20] Wang G , Xu J , Qi Y , Xiu J , Li R , Han M . Distribution of brain metastasis from lung cancer. Cancer Manag Res. 2019;11:9331‐9338. 31802951 10.2147/CMAR.S222920 PMC6830371

[21] Wang Y , Xia W , Liu B , et al. Exploration of spatial distribution of brain metastasis from small cell lung cancer and identification of metastatic risk level of brain regions: a multicenter, retrospective study. Cancer Imaging. 2021;21:41. 34120659 10.1186/s40644-021-00410-w PMC8201893

[22] Hayashida Y , Hirai T , Morishita S , et al. Diffusion‐weighted imaging of metastatic brain tumors: comparison with histologic type and tumor cellularity. AJNR Am J Neuroradiol. 2006;27:1419‐1425. 16908550 PMC7977525

[23] Duygulu G , Ovali GY , Calli C , et al. Intracerebral metastasis showing restricted diffusion: correlation with histopathologic findings. Eur J Radiol. 2010;74:117‐120. 19359117 10.1016/j.ejrad.2009.03.004

[24] Zulfiqar M , Yousem DM , Lai H . ADC values and prognosis of malignant astrocytomas: does lower ADC predict a worse prognosis independent of grade of tumor? A meta‐analysis. AJR Am J Roentgenol. 2013;200:624‐629. 23436853 10.2214/AJR.12.8679

[25] Poussaint TY , Vajapeyam S , Ricci KI , et al. Apparent diffusion coefficient histogram metrics correlate with survival in diffuse intrinsic pontine glioma: a report from the pediatric brain tumor consortium. Neuro Oncol. 2016;18:725‐734. 26487690 10.1093/neuonc/nov256 PMC4827042

[26] Raso MG , Bota‐Rabassedas N , Wistuba II . Pathology and classification of SCLC. Cancer. 2021;13:820. 10.3390/cancers13040820 PMC7919820 33669241

